# Evaluation of headstarting as a conservation tool to recover Blanding’s Turtles (*Emydoidea blandingii*) in a highly fragmented urban landscape

**DOI:** 10.1371/journal.pone.0279833

**Published:** 2023-03-08

**Authors:** Tharusha Wijewardena, Matthew G. Keevil, Nicholas E. Mandrak, Andrew M. Lentini, Jacqueline D. Litzgus

**Affiliations:** 1 School of Natural Sciences, Laurentian University, Sudbury, Ontario, Canada; 2 Department of Biology, University of Toronto, Toronto, Ontario, Canada; 3 Toronto Zoo, Scarborough, Ontario, Canada; Bowling Green State University, UNITED STATES

## Abstract

Freshwater turtle populations are declining globally as a result of anthropogenic activities. Threats to turtles in urban areas are exacerbated by road mortality and subsidized predators, which can lead to catastrophic shifts in population size and structure. Headstarting is used as a conservation tool to supplement turtle populations that may otherwise face extirpation. A headstarting program began in 2012 to recover a functionally extinct population of Blanding’s Turtles (*Emydoidea blandingii*) 26in Rouge National Urban Park (RNUP), Ontario, Canada. The original population included five adults and one juvenile turtle. From 2014 to 2020, 270 headstarted turtles were released. The population has been monitored annually since 2014 using visual-encounter surveys, radio-telemetry, and live trapping (from 2018 onwards). We used mark-recapture and radio-telemetry data to quantify abundance, survival, and sex ratio of the headstarted turtle population. Using a Jolly-Seber model, we estimated abundance to be 183 turtles (20 turtles/ha) in 2020. Estimated survival of headstarted turtles approached 89%, except for turtles released in 2019 when survival was 43% as a result of a known mass mortality event at the study site. Pre- and post-release sex ratios were not significantly different (*χ*^2^ = 1.92; p = 0.16), but shifted from 1:1.5 to 1:1 male:female post-release. Given that the headstarted turtles have not yet reached sexual maturity, it is unclear whether headstarted turtles will reach adulthood and successfully reproduce to maintain a self-sustaining population. Thus, to evaluate the success of the headstarting program, long-term monitoring is required.

## Introduction

Freshwater turtles are facing global declines resulting from anthropogenic activities including habitat fragmentation, degradation and loss, introduction of invasive species, environmental pollution, diseases and parasitism, overexploitation, and the global climate crisis [1–3]. Turtles in urban habitats experience additional threats, such as road and rail mortality, subsidized predators, and reduced nesting success, which may directly or indirectly lead to biased population structures that result in functional extinction of a population long before extirpation [4–6]. The bet-hedging life-history strategy of turtles (i.e., long generation time, late sexual maturity, high mortality rate of eggs and juveniles) further limits population recovery, making them one of the most threatened vertebrate groups with 61% of turtle species at risk of extinction [6, 7]. Given the magnitude and scope of threats affecting all life-history stages of turtles, recovery of turtle populations has become increasingly difficult [3].

Several conservation interventions have been used to mitigate the decline of turtle populations. Captive breeding, establishing genetic-assurance populations, reintroductions, relocations, and removal of invasive species have prevented extirpation of several turtle populations in the short-term [8]. Recently, headstarting has become popular as a reintroduction strategy to augment at-risk turtle populations [9]. For turtles, headstarting typically involves the collection of eggs from the wild, then incubation and rearing of hatchlings for 1–4 years in human care, and subsequent release to the wild [9, 10]. Headstarting aims to increase the survival of hatchlings by raising them in human care during their most vulnerable stages, thereby promoting increased somatic growth rate, larger body size [11], and earlier age at sexual maturity compared to wild conspecifics of similar age [10, 12].

Although headstarting is becoming an increasingly common conservation tool, it has been criticized as a halfway measure or a distraction from more effective conservation actions such as the protection of adult turtles [13]. To ensure headstarting is more than a halfway measure, root causes of population decline need to be addressed; however, many conservation agencies lack sufficient resources to implement recovery strategies that adequately address the root causes [9, 14]. Although turtle population persistence is more sensitive to adult survivorship than to hatchling and juvenile survivorship [15, 16], population models based on freshwater turtles in northern latitudes have indicated that juvenile survival may play an important role in population persistence when improving already high adult survival is impractical [17, 18]. Additionally, many headstarting programs are relatively new, with each reporting variable methods, sample sizes, outcomes [8], and challenges [10, 19], making it difficult to evaluate headstarting as a viable reintroduction strategy to recover turtles at a local scale. Despite the on-going debate, headstarting remains a popular tool among conservation practitioners.

A functionally extinct population of Blanding’s Turtle (*Emydoidea blandingii*) was identified in the Rouge National Urban Park (RNUP) in Toronto, Ontario, Canada. The RNUP is a protected area situated in a metropolis (approximately 2.8 million people [20]) with an extensive network of roads and railways, rapid urban development, and high levels of habitat degradation and fragmentation [21, 22]. More than 85% of the historical wetland areas in Toronto have been lost to urban developments and agriculture [23] and over 75% of the park area has been altered by human-induced disturbances [24]. Although the root causes of the population decline of Blanding’s Turtles in the RNUP have not been clearly identified, the highly urban nature of the study site likely contributed to the loss of turtles. Surveys from 2005 to 2006 in the RNUP and adjacent wetlands found six Blanding’s Turtles (three males, two females, one juvenile) and an opportunistic sighting of a road-killed nesting female (Toronto Zoo [Unpublished]). Given the degree and rate of habitat modifications and human-induced threats that persist in urban environments like Toronto, it was expected that the Blanding’s Turtle population would be extirpated within a few years without human intervention. As a result, the Toronto Zoo, Parks Canada, and other conservation partners initiated a headstarting program in 2012 to prevent extirpation of the turtle population. Given the limited resources available for conservation in general and the funding allocated over the past 10 years to restore the Blanding’s Turtle population in the RNUP, it is critical to evaluate headstarting to justify continued support for the program. Headstarting success depends on the high survival of headstarted turtles in human care and after release, thereby increasing the long-term abundance of turtles in the wild [25]. In addition, to ensure the long-term persistence of a population, it is important to maintain stable population structures especially in urban environments where turtles are susceptible to male-biased population sex ratios through differential mortality of females[5, but see 26]. The goal of our work was to quantify: (1) population size (i.e., abundance) and density; (2) survival; and (3) sex ratio of the headstarted population using mark-recapture and radio-telemetry data. By quantifying these short-term indicators of success, we provide an early evaluation of whether headstarting is a viable conservation tool to recover the Blanding’s Turtle population in the RNUP. We also provide a snapshot of the population demographics that can be used as a baseline for future analyses about population vital rates, not only in the RNUP but also for freshwater turtles in other urban landscapes that are likely to face similar challenges.

## Methods

### Study site and history

**Fig 1:**
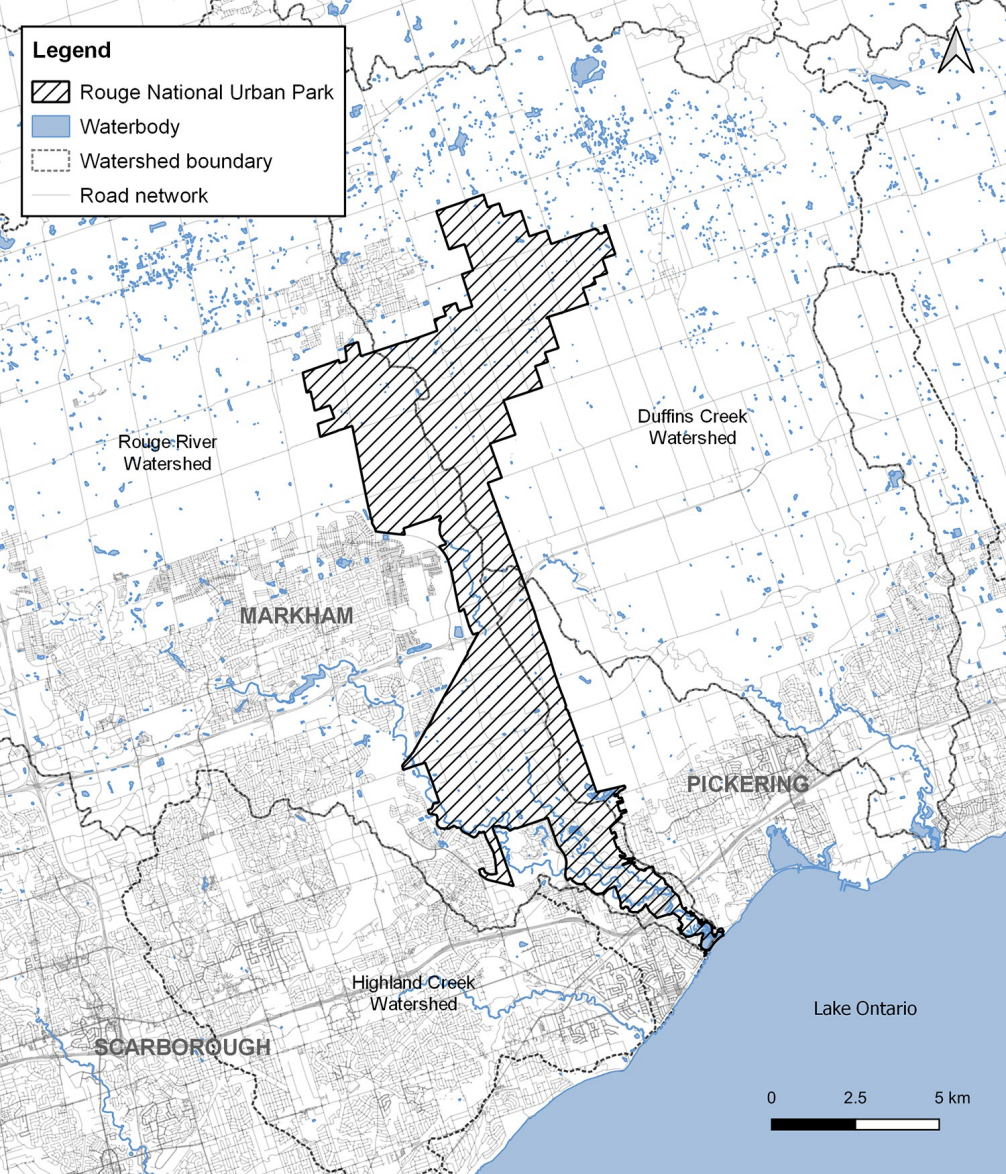
Spatial overview of the Rouge National Urban Park in Toronto, Ontario, Canada. The exact location of the study site is excluded to protect at-risk species from poaching. The map was created by compiling data available from https://geohub.lio.gov.on.ca/ on QGIS (3.20.1-Odense) [29–32].

We conducted our research at the RNUP in Toronto, Ontario, Canada (43.8188° N, 79.1728° W). The RNUP is the first urban national park in Canada and is part of a pilot project carried out by Parks Canada to conserve urban biodiversity, Indigenous cultural landscapes, and agricultural heritage of the area [27]. It is an ecologically protected zone established in 2015 under the Rouge National Urban Park Act [28] that encompasses 80 km^2^ of forests, meadows, rivers, wetlands, and fragments of rare habitats such as oak savannah and Carolinian woodlands [27]. Situated at the center of the Canada’s largest metropolitan area (Fig 1), the park is surrounded by major highways, freight and passenger railways, residential, commercial, and industrial developments, and agricultural lands [27].

Our study site is situated in the southern portion of the RNUP. In the early 1990s, the area was restored to a wetland complex of vernal pools, and more permanent ponds of various sizes with littoral vegetation including alders (*Alnus* spp.), cattails (*Typha* spp.), sedges (*Carex* spp.), and willows (*Salix* spp.) [21]. More recently, invasive species, such as European common reed (*Phragmites australis*), garlic mustard (*Alliaria petiolate*), purple loosestrife (*Lythrum salicaria*), and reed canary grass (*Phalaris arundinacea*) have become ubiquitous. Once restoration efforts were completed, the Toronto Zoo’s Adopt-A-Pond Wetland Conservation Program began wetland surveys to evaluate species occurrence in the area. The surveys found three at-risk turtle species: Painted Turtle (*Chrysemys picta*), Snapping Turtle (*Chelydra serpentina*), and the globally endangered [33] Blanding’s Turtle. In Canada, Painted and Snapping turtles are designated as ‘Special Concern’ by the Committee on the Status of Endangered Wildlife in Canada (COSEWIC) [34, 35], and Blanding’s Turtle is designated as ‘Endangered’ [36].

In 2005, the Blanding’s Turtle population within the park boundary was known to be comprised of three adult turtles (two males and one female) and a juvenile. Two additional adult turtles (one male and one female) were discovered in 2006 in an adjacent creek approximately 4 km from the RNUP (Toronto Zoo [Unpublished]). Given that the Blanding’s Turtle population in the RNUP was presumed functionally extinct, the Toronto Zoo initiated a headstarting program in 2012 to supplement the wild population [21]. A preliminary population viability analysis (PVA) showed that 40 headstarted turtles with 1:1.5 male:female sex ratio would need to be released each year for 20 years to reach a self-sustaining population of 150 adult Blanding’s Turtles (Toronto Zoo [Unpublished]). The first release occurred in 2014 with 10 juveniles, followed by 21 in 2015, 36 in 2016, 49 in 2017, 49 in 2018, 48 in 2019, 57 in 2020 for a total of 270 headstarted turtles released to date (Toronto Zoo [Unpublished]). An additional 184 hatchlings were released without headstarting because the number of eggs that hatched exceeded the capacity of the Toronto Zoo rearing facility.

### Headstarting protocol

Since 2012, the Toronto Zoo team and partners have collected Blanding’s Turtle eggs from wild populations across Ontario. Each year, approximately 10–150 eggs are collected and incubated ex-situ using standard protocols (available upon request). Blanding’s Turtles have temperature-dependent sex determination, and males are produced when the eggs are incubated at or below 28°C and females are produced at incubation temperatures above 30°C [37, 38]. At the Toronto Zoo, eggs are incubated at 27.5°C and 29.5°C to yield a 1:1.5 male:female sex ratio. Annual hatching success at the Toronto Zoo has ranged from 72% to 100%.

Hatchlings are reared in groups for two years prior to release. A maximum of 15 hatchlings are housed in each Waterland black plastic tub (91 cm x 45 cm x 40 cm), with shallow water (20 cm) and artificial vegetation for one year. After a year, a maximum of 7 hatchlings are housed in each tub. The water temperature is maintained at 25–27˚C in the first year and 23–24˚C in the second year. A 180 L sump filter with a heater located below the tanks is used for water circulation. The filter is cleaned once a week and the water is changed three times a week. One end of the tub is elevated and lined with rocks and pebbles, and a 50W bulb provides a basking area with 28–35˚C. A ramp is placed to facilitate easy access to the basking area by hatchlings. Two fluorescent UVB bulbs with full-spectrum lighting are provided for proper bone growth. The headstarted turtles are fed three times a week at roughly 5% of the average body mass of the cohort. One-year old hatchlings are fed turtle gel and beef heart gel (gel diets are gelatine-based foods for aquatic species formulated by the Toronto Zoo), earthworms, and romaine lettuce. After a year, all turtles are supplemented with fish (smelt) and live crickets. As part of enrichment and to promote better foraging ability in the wild, turtles are given varied food sizes, live worms, and natural tree bark for cover.

A maximum of 60 turtles are reared in human care for two years at the Toronto Zoo and released in June each year. A month before their release, headstarted turtles are relocated to large outdoor tubs (173 cm x 120 cm x 58 cm) with shallow water (25 cm) and artificial vegetation. Each outdoor tub holds a maximum of 25 turtles, and the number of tubs used varies annually. The water temperature in outdoor tanks is maintained at 28°C. Each tub is equipped with a filter and a water pump to create a current. The outdoor holding area is secured using mesh and roof fencing. All headstarted turtles are weighed using an Ohaus CS-series scale to the nearest 0.1 g and measured monthly for two years using Belt-Art calipers to the nearest 0.1 mm. Standard body measurements are recorded including midline carapace length and width, midline plastron length and width, shell height, and body mass. Prior to release, turtles are individually marked with notches on the marginal scutes [39] and a subcutaneous PIT (passive integrated transponder) tag is inserted into the left hind leg.

### Mark-recapture surveys

We conducted mark-recapture surveys in the RNUP from 2018 to 2020 during the turtle active season (May to mid-September). We deployed 5–10 baited hoop traps and a basking trap (in 2018 only) to capture turtles. We also included opportunistic hand captures to maximize sample size. Hoop traps were baited with sardines or cat food. Both hoop traps and basking traps were checked daily for turtles. Upon capture, we recorded standard morphological measurements including midline carapace length and width, plastron length and width, height (using calipers [ZEAST Vernier] to the nearest 0.1 mm) and body mass of the turtles (using a Pesola scale to the nearest 1 g). We calculated the sampling effort for each year by calculating the number of trap days. Trap days were quantified by multiplying the number of traps by the number of days the traps were deployed. The sampling effort was 445 trap days in 2018, 570 in 2019, and 410 in 2020.

### Radio-telemetry surveys

We conducted radio-telemetry surveys from 2014 to 2019. During the turtle active season, from May to mid-September, we tracked turtles two to three times weekly, whereas in the non-active season, we tracked once monthly. Each year, a subset of headstarted turtles were outfitted with radio transmitters (Advanced Telemetry Systems R1600) and tracked for at least one year. To determine which turtles would receive a radio transmitter, headstarted turtles from each cohort were separated into two groups based on presumed sex (i.e., incubation temperature). Each turtle was assigned a unique number and using the random number function (= RAND) in Excel, a turtle was selected to be outfitted with a radio transmitter. Each year, more female turtles were selected for radio transmitter attachment. The combined weight of the transmitter and the epoxy was approximately 10 g, which was less than 10% of the average turtle body mass. Headstarted turtles were tracked using an R410 or R4000 Receiver (Advanced Telemetry Systems, Inc., MN). We tracked 10 turtles from the 2014 release cohort, 21 turtles from 2015, 24 from 2016, 16 from 2017, 22 from 2018, and 23 from the 2019 release cohort. We excluded the 10 individuals from the 2014 release cohort in our analysis because of missing data. Most turtles were only tracked for a year, but a few headstarted turtles remained in the study to be radio-tracked for 2–5 additional years (Table 1). The turtles that remained in the study were affixed with a new radio transmitter each year.

**Table 1:** Number of headstarted Blanding’s Turtles included in the Jolly-Seber open population model. Headstarted turtles were grouped based on release year (release cohort) and further subdivided based on number of years tracked, which resulted in 23 groups.

Release cohort	Tracking duration (# of occasions)	Number of turtles	Total released
2015	6	3	21
	5	4	
	4	2	
	2	11	
	1	1	
2016	5	5	36
	4	4	
	3	1	
	2	8	
	1	6	
	0	12	
2017	4	2	49
	3	4	
	2	10	
	0	33	
2018	3	2	49
	2	19	
	1	1	
	0	27	
2019	2	18	48
	1	5	
	0	25	
2020	0	57	57

### Ethics statement

All use of animals in this study was approved by the Toronto Zoo (Ref. No. 2010-01-01, 2014-03-01, 2015-04-01, 2017-03-01, 2020-02-01) and Laurentian University (protocols AUP 2017-02-01 and 6020983) Animal Care Committees. All work was authorized by Ministry of Northern Development, Mines, Natural Resources, and Forestry (NDMNRF) Scientific Collector’s Authorizations (1077386, 1080550, 1083631, 1086727, 1089107/Local Ref. No. AU2018-0533, 1092095/Local Ref. No. AU2019-1299, and 1095690/Local Ref. No. AU2020-2325), Endangered Species Act permits (AU-B-010-14, AU-B-008-15, AU-B-011-16, AU-B-008-17, AU-B-009-18), and Parks Canada Species at Risk Act permits (RNUP-2020-35017). We obtained informed written consent from all organizations.

### Statistical analyses

#### Abundance and survival estimates

**Fig 2:**
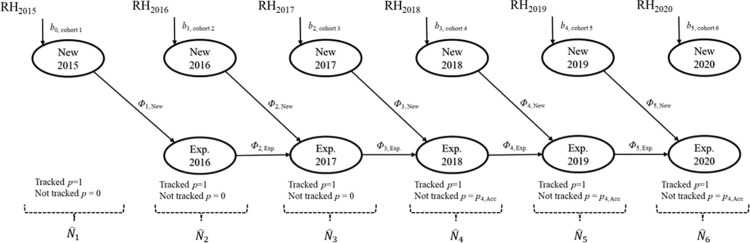
Simplified conceptual framework of the Jolly-Seber open population model used to estimate abundance and survival of headstarted Blanding’s Turtles in the Rouge National Urban Park. The analysis included 260 released headstarted turtles, of which 139 had capture histories from mark-recapture and radio-telemetry surveys. Survival and detection parameters were indexed by time and by acclimation (acc), which had two levels: ‘New’, for newly released turtles and Exp. (experienced) after more than one year in the wild. Probability of entry (*b*) was indexed by time and release cohort. *b*_0_ occurs prior to the first sampling occasion. RH_year_ refers to the size and year of each release. ${\hat{N}}_{t}$ refers to population size in each year. Implementation of this model included partitioning of release cohorts into smaller groups according to telemetry duration so that detection probability, *p*, could be fixed at 1 on specific occasions when turtles were tracked.

We used the Jolly-Seber POPAN formulation [40] open population model in Program MARK [41] to obtain annual abundance $\left(\hat{N}\right)$, apparent survival (*Φ*) and recapture probability (*p*) estimates of the headstarted turtle population. The POPAN Jolly-Seber model includes net recruitment (i.e., the number of new individuals entering the population), which is useful for parameterizing the annual release of headstarted turtles. We used data from mark-recapture (2018–2020) and radio-telemetry surveys (2015–2019), which resulted in 139 unique headstarted turtles with capture histories. Radio-telemetry data included 106 turtles that were tracked 1–5 years; mark-recapture surveys and opportunistic sightings yielded 33 additional turtles. We did not include five headstarted turtles that were opportunistically caught 2015–2017 because we conducted mark-recapture surveys only from 2018 to 2020. We also excluded three headstarted turtles that were found dead at first encounter 2018–2019. We excluded the 10 individuals released in 2014 from the analysis because of missing data. All turtles used in this analysis were from the 2015–2020 release cohorts. We did not include 184 direct-release hatchlings (i.e., non-headstarted turtles) in our analysis because only one was ever recaptured during the analysis period, and because our goal was to quantify the demographic contribution of the headstarted population. Given that the turtles in our dataset were from different release years and tracked for different durations, we grouped turtles based on release year (i.e., release cohort) and subdivided each release cohort by the number of years tracked; this resulted in 23 groups (Table 1). Grouping enabled us to parameterize the known number and timing of new entrants to the headstarted population (i.e., annual release event) by fixing the probability of entry in each year (*b*_*t*_) and the superpopulation parameter (total number of individuals ever alive) for each group. To parameterize new entrants to the population, we fixed the initial population size for each cohort group (which is the same as the group superpopulation in our application) as the number of headstarted turtles released. We fixed the probability of entry to specify the timing of release for each cohort group by fixing *b*_*group*,*t-1*_ = 1 for a cohort group released at time *t*; otherwise, we fixed *b*_*group*,*t*_ = 0 (Fig 2).

**Fig 3:**
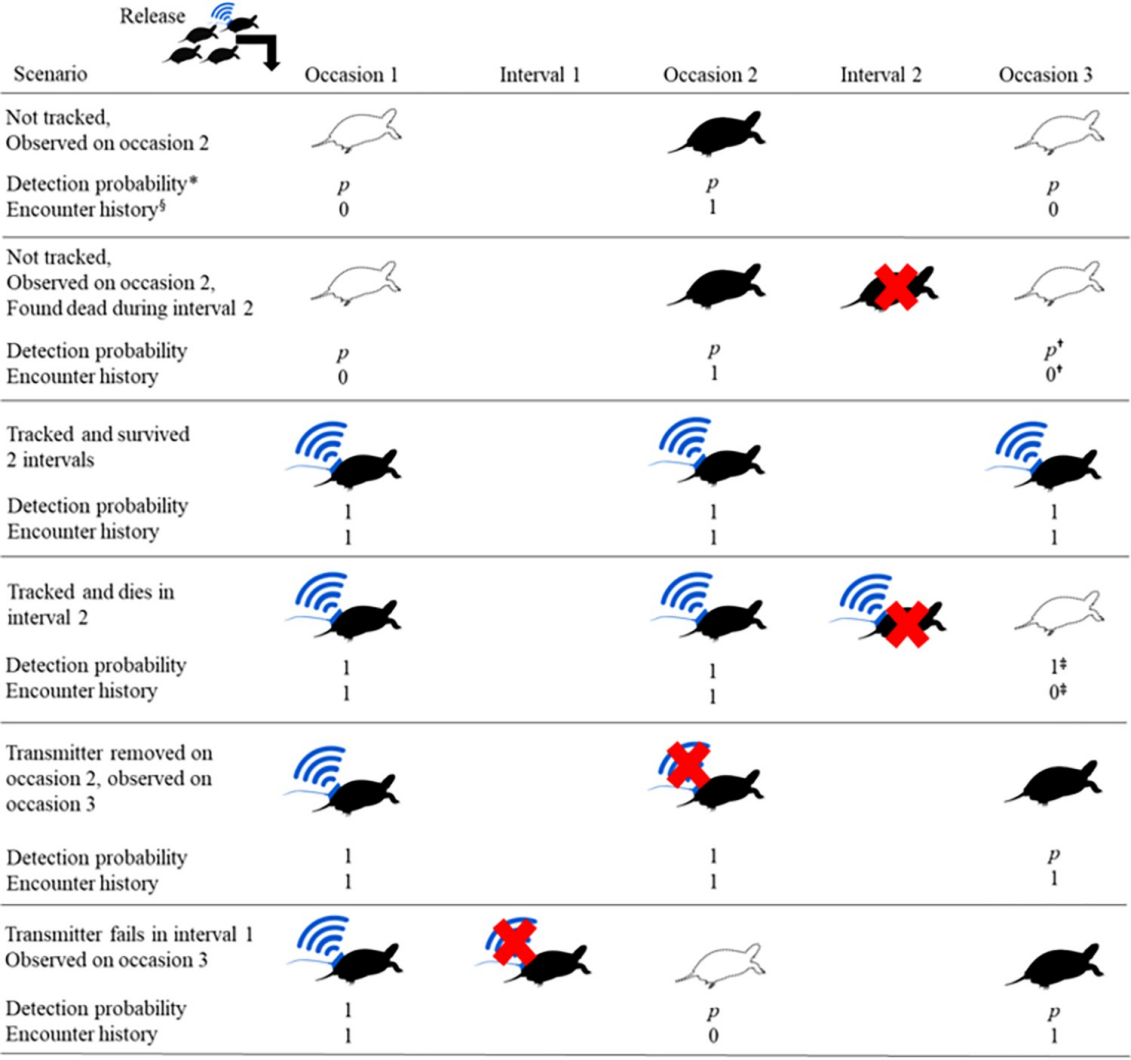
Conceptual diagram of how survival, telemetry, and detection were represented in encounter histories and using parameter fixing of detection probability, *p*, for a subset of potential scenarios. Scenarios are illustrated for a single Blanding’s Turtle release cohort over three sets of annual surveys. Hollow turtle symbols indicate an individual was not observed, a solid turtle symbol indicates that the turtle was observed. The red X indicates mortality, and the blue “wifi” symbol indicates a radio transmitter. Sampling by trapping and opportunistic detection were collapsed into one occasion per year with survival occurring during intervals between occasions. Telemetry observations of live individuals in May were considered live encounters during the corresponding annual occasion; otherwise, we considered telemetry observations during the rest of the year to occur during intervals, which are not represented directly in encounter histories. *Detection probability is conditioned on survival; ^**†**^Jolly Seber models use only live encounter data, even when mortality is observed; ^§^Encounter history is a binary sequence: 1 when an individual is observed alive, 0 when not observed; ^**‡**^Conditional on being released, turtles are encountered according to the product of current detection probability and previous survival. Certain detection (*p* = 1) but no encounter, implies no survival over a previous occasion, which informs survival estimation.

To accommodate radio-telemetry data in the mark-recapture model, we fixed the recapture probability at 1 when headstarted turtles were tracked because telemetered turtles were always detectable. We fixed the recapture probability from 2015 to 2017 at 0 for individuals without radio transmitters because mark-recapture surveys only occurred from 2018 to 2020. For all headstarted turtles, the recapture probability prior to release was fixed at 0 because turtles were not available to be captured in the wild. Other scenarios were coded according to Fig 3.

Candidate models of apparent survival and recapture probability included full time-dependence, constant probability (null model), and acclimation, which had two levels, one for newly released turtles (New), and one for subsequent years (Exp.). A recent study on headstarted Blanding’s Turtles described acclimation period as a one-to-two-year interval during which headstarted turtles may exhibit different behaviours from wild ones due to inexperience in their release environment [42]. We hypothesized that the headstarted turtles in our population may exhibit different behaviours in the first year of their release that affect their apparent survival and recapture probability. We also investigated whether there was a cohort effect on apparent survival. We defined our models based on biological relevance and a priori hypotheses that reflected headstarting protocols and variation in annual survey effort. In MARK, we specified a logit link function for apparent survival and recapture parameters and a multinomial logit link function for probability of entry, and a log link function to estimate the population size for each group at each occasion. We ranked the relative support for each model based on Akaike’s Information Criterion for small sample size (AIC_c_) [43]. We assessed the goodness of fit by examining the Fletcher $\hat{c}$ value for the general model, which included full time-dependence and acclimation effects for both apparent survival and recapture probability. To calculate population size based on the best model, we first obtained the abundance estimate for each cohort by summing the abundance estimates of each telemetry group (i.e., tracked for 0–5 years) in the cohort. We then summed the abundance estimates from all release cohorts (6 cohorts) to obtain the total population size in year 2020. The confidence intervals for abundance estimates were calculated using the Delta method [44].

#### Sex ratio

We used a χ^2^ test to evaluate whether there was a significant difference between the pre-release and post-release sex ratio of headstarted turtles in the RNUP population using R (version 4.1.2) [45]. The pre-release male:female sex ratio was 1:1.5, based on egg incubation temperature. To calculate post-release sex ratio, we used data from mark-recapture surveys from 2018 to 2020. Marginal scute notches and PIT tags were used to identify each turtle. We pooled all the turtles captured between 2018 and 2020, which resulted in 46 headstarted turtles. If a turtle was captured multiple times, only the first capture was included in the analysis. Sex of each headstarted turtle was presumed based on pre-recorded egg incubation temperature data. Given that none of the headstarted turtles have reached maturity, secondary sexual characteristics could not be used to determine sex.

## Results

### Abundance and survival estimates

**Fig 4:**
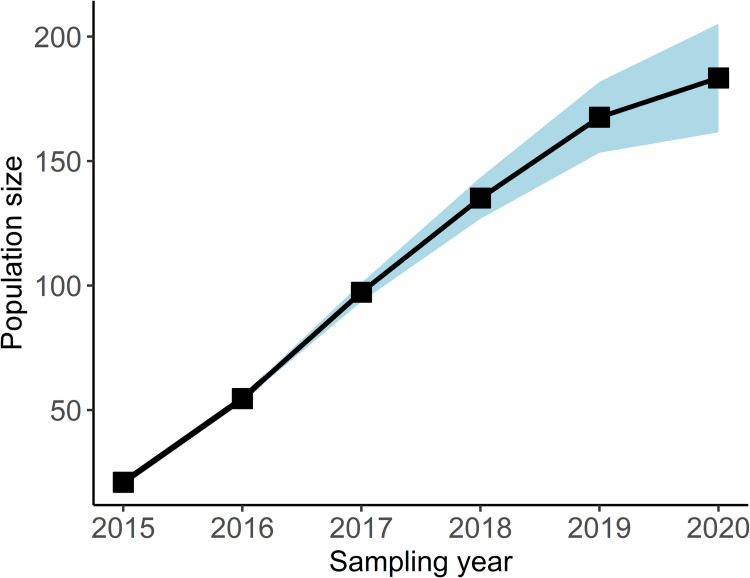
Population size of headstarted Blanding’s Turtles in the Rouge National Urban Park estimated using the Jolly-Seber open population model. The blue shading indicates the 95% confidence interval for population size.

**Fig 5:**
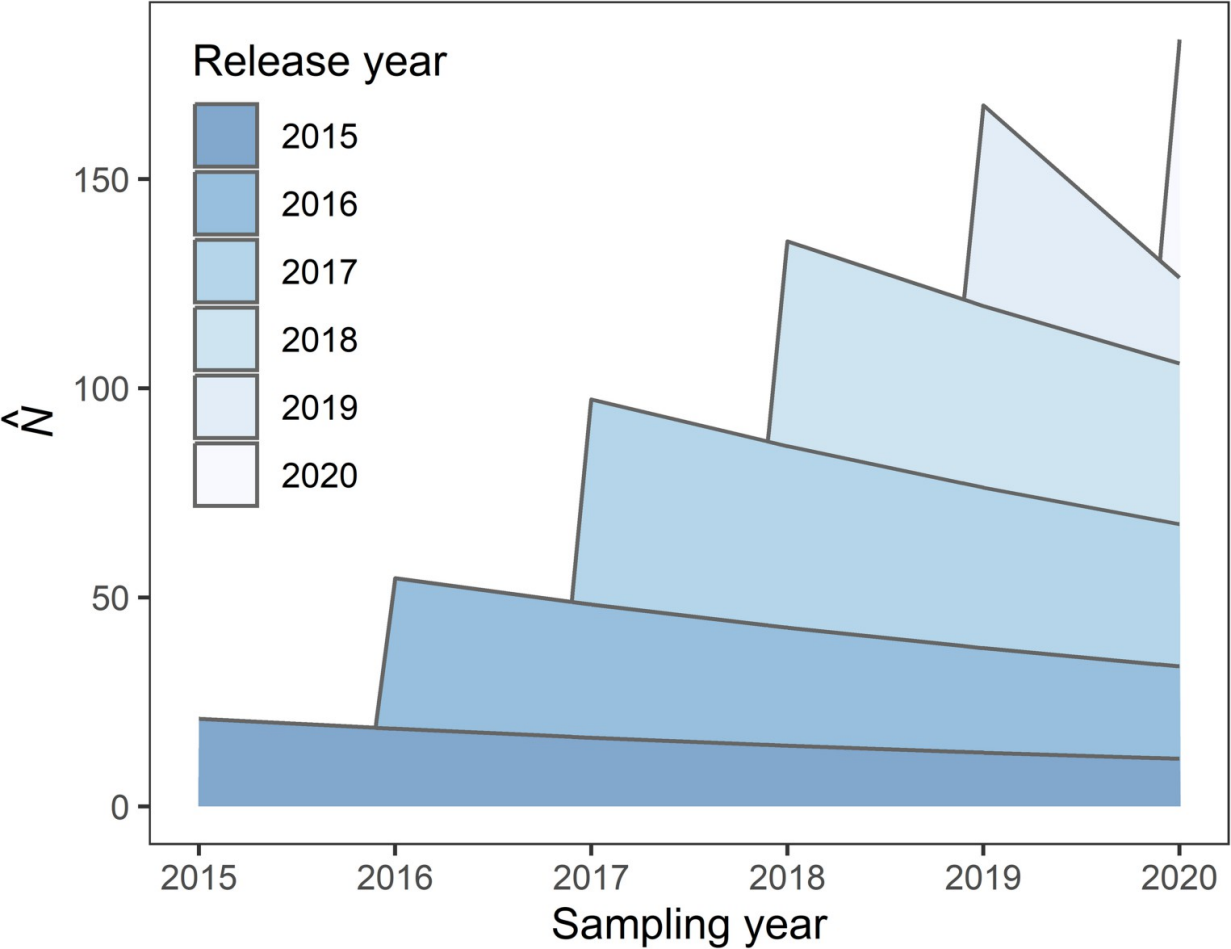
Headstarted Blanding’s Turtle abundance in the Rouge National Urban Park based on release year. The population size ($\hat{N}$) increased each year but the number of turtles in each cohort decreased each year as a result of mortality. The total abundance in 2020 was 218 headstarted turtles.

A total of 270 headstarted turtles were released into the RNUP, of which 139 were recaptured or tracked by telemetry. Excluding telemetry relocations, we recaptured 19 headstarted turtles in 2018, 21 in 2019, 13 in 2020. The top model of our initial candidate set was *Φ*_t*acc_ *p*_t_, which had 12 parameters, although several of these could not be estimated. Inestimable parameters often occur within models that are overparameterized for a given dataset. Under this model, survivorship was either inestimable or similar among combinations of time and acclimation level with one exception: survivorship was much lower for newly released headstarts in 2019. Of the 18 newly released headstarted turtles tracked for the full interval from June 2019 to May 2020, 10 were found dead in that period, while 17 tracked turtles died over all four of the preceding intervals. This is also higher than the two mortalities observed among the 12 turtles from older cohorts tracked during 2019–2020. This single year/acclimation effect could only be separately accounted for by survivorship sub models *Φ*_t*acc_ or *Φ*_cohort_. Cohort-survival models were not favoured by model selection (Table 2) and *Φ*_t*acc_ requires many parameters to estimate survivorship separately for each combination of time and acclimation level. Therefore, we proposed a set of *post hoc* models to be added to our candidate set, which would allow survival to differ for 2019 headstarted turtles but with fewer parameters than the full *Φ*_t*acc_ model. The survival component of these models included a time effect for each interval and a 2019 acclimation effect *Φ*_t + acc2019_, an acclimation effect and a 2019 time effect *Φ*_acc + t2019_, or constant survival except for new releases in 2019 *Φ*_acc2019_. We also examined a model, *Φ*_2019_ that allowed survival to differ in the 2019 sampling year only. In combination with detection sub models, this resulted in 18 *post hoc* models being added to the candidate set (Table 2). Multiple models had ΔAIC_c_ <2 and similar deviance values, indicating similar levels of support to the top model [43]. Following Arnold 2010 [46], we selected a preferred model *Φ*_acc2019_*p*_t_ with the fewest parameters and rejected those that used additional parameters without a substantial improvement in deviance as quantified by the AICc score. Based on the best supported model *Φ*_acc2019_*p*_t_ (Table 2), overall abundance increased each year as new headstarted turtles were released. We estimated turtle abundance in 2020, at the end of the study period, to be 183 turtles (95% CI = 162–205) (Figs 4 and 5). With 183 individuals occupying 9 ha wetland complex, the density of headstarted Blanding’s Turtles in our study area is 20 turtles/ha.

**Table 2:** Summary of Jolly-Seber POPAN model ranking. *Φ* and *p* refer to apparent survival and recapture probabilities, respectively for Blanding’s Turtles in the Rouge National Urban Park. ‘acc’ refers to the effect of acclimation with two levels: newly released headstarted turtles and those in their first year following release.

Model	*k*	AICc	ΔAICc	*w*	Deviance
*Φ*(t+acc2019)*p*(t) ^§^	9	3089.93	0.00	0.18	2894.26
*Φ*(t+acc2019)*p*(t*acc) ^§^	12	3090.83	0.90	0.11	2888.67
*Φ*(t+acc2019)*p*(t+acc) ^§^	12	3091.24	1.32	0.09	2889.08
*Φ*(acc2019)*p*(t) ^§^	5	3091.60	1.67	0.08	2904.37
*Φ*(acc + t2019)*p*(t) ^§^	6	3091.83	1.90	0.07	2902.51
*Φ*(acc + t2019)*p*(t*acc) ^§^	9	3091.89	1.96	0.07	2896.22
*Φ*(acc + t2019)*p*(t+acc) ^§^	9	3091.89	1.97	0.07	2896.23
*Φ*(t*acc)*p*(t)	12	3092.01	2.09	0.06	2889.85
*Φ*(acc2019)*p*(t*acc) ^§^	8	3092.07	2.14	0.06	2898.53
*Φ*(acc2019)*p*(t+acc) ^§^	8	3092.22	2.29	0.06	2898.69
*Φ*(cohort)*p*(t)	8	3092.98	3.05	0.04	2899.44
*Φ*(t*acc)*p*(t*acc)	15	3093.29	3.36	0.03	2884.48
*Φ*(cohort)*p*(t*acc)	11	3093.79	3.87	0.03	2893.81
*Φ*(cohort)*p*(t+acc)	11	3093.90	3.98	0.02	2893.92
*Φ*(t)*p*(t)	8	3094.85	4.92	0.01	2901.31
*Φ*(t)*p*(t*acc)	11	3095.01	5.08	0.01	2895.03
*Φ*(t)*p*(t+acc)	11	3095.63	5.70	0.01	2895.64
*Φ*(2019)*p*(t*acc) ^§^	8	3098.23	8.31	0.00	2904.70

§, *post hoc* models that were proposed based on initial results suggesting a specific 2019 acclimation effect on *Φ; k*, number of parameters; AIC_c_, Akaike’s Information Criterion with small sample correction; ΔAIC_c_, difference in AIC_c_ between a given model and the best supported model; *w*, Akaike weight

The mean carapace length of headstarted turtles at hatching was 36.3 mm [CI = 36.1–36.5, n = 286, SD = 1.8] and mean body mass was 10.2 g [CI = 10.0–10.3, n = 286, SD = 1.3] whereas at release, mean carapace length was 95.5 mm [CI = 94.8–96.2, n = 270, SD = 6.1] and mean body mass was 169.8 g (CI = 166.2–173.4, n = 270, SD = 29.9). Population structure was juvenile-biased given that only six wild adults have been observed in the park and that majority of the population is comprised of released headstarted turtles.

Based on the best model (*Φ*_acc2019_*p*_t_), apparent survival remained high and constant for turtles released in 2015–2018 across all sampled years, but the turtles released in 2019 had a considerably lower survival estimate in the release year. The annual survival of headstarted turtles was 0.89 (95% CI = 0.82–0.93) from 2015 to 2018, and 0.43 (95% CI = 0.23–0.65) for the 2019 cohort in the release year. The recapture probability was influenced by sampling year. The recapture probability was 0.34 (95% CI = 0.24–0.45) in 2018, 0.10 (95% CI = 0.06–0.17) in 2019, and 0.04 (95% CI = 0.02–0.09) in 2020.

#### Sex ratio

We investigated whether the post-release sex ratio significantly differed from the pre-release sex ratio. Pooling the data from three years, and including only the first capture of each turtle, resulted in 46 unique headstarted turtles, of which 23 were presumed males and 23 were presumed females (based on incubation temperature). This apparent decrease in the proportion of females resulted in a male:female sex ratio of 1:1, and was not significantly different from the pre-release sex ratio of 1:1.5 (*χ*^2^ = 1.92; P = 0.16).

## Discussion

Our results indicate successful initial steps towards recovery of the Blanding’s Turtle population in the RNUP because abundance has increased over the course of the headstarting program, population sex ratio is balanced, and individual headstarted turtles experienced high survival post-release. One exception to this is the 2019 release cohort that experienced unusually high mortality in the release year, likely from a mass mortality event that occurred in the study area. Given the goal of headstarting is to establish a self-sustaining population, and given the on-going challenges in the study area (e.g., subsidized predators, habitat degradation), long-term monitoring is required to fully assess whether headstarting is a viable conservation tool to recover Blanding’s Turtles in the RNUP.

### Abundance and survival estimates

In 2020, the estimated population size of headstarted Blanding’s Turtles in the RNUP reached 183 juvenile turtles with a density of 20 turtles/ha. Our density estimates are one of the highest compared to population densities reported elsewhere (Table 3). However, the majority of available density estimates are based primarily on observations of adults, which is likely to substantially underestimate the total number of post-hatching individuals. Low detection of juveniles is a widespread problem in surveys of Blanding’s Turtles [15] and other reptiles [47]. Congdon et al. [15] estimated a life table for Blanding’s Turtles in Michigan, and we calculated that the corresponding Leslie matrix implies a stable stage distribution in which juveniles more than one year old outnumber adults with a ratio of 4.3:1 [48, 49]. Therefore, direct comparison between the density of headstarted juveniles at the RNUP to other published values should be made cautiously, given that juvenile abundances are typically neglected or underestimated. In addition, density estimation is sensitive to size of the area in the study site deemed suitable habitat for the turtles. For example, Zagorski, Boreham and Litzgus [50] reported that the density was calculated by dividing population size by area of wetlands where turtles were found, excluding deep water (>5 m), nesting habitats, and terrestrial habitats. In our analysis, the 9 ha wetland complex included waterbodies of all depths and adjacent land areas because juvenile turtles have been observed in all ponds and on land while basking or aestivating (located via radio-telemetry). If we calculated density similar to Zagorski, Boreham and Litzgus [50], then our density estimate would be higher. Our findings suggest that juvenile Blanding’s Turtles can tolerate high densities in an urban environment.

**Table 3:** Densities of adult Blanding’s Turtle in populations from Canada and the USA.

Location	Density (turtles/ha)	Source
Ontario, Canada	0.12–1.84	[36, 50]
Nova Scotia, Canada	0.05–0.3	[36]
Minnesota, USA	0.35–1.45	[51, 52]
Michigan, USA	8.8–10	[53]
Maine, USA	3.9–5.9	[54]
Wisconsin, USA	27.5	[55]
Missouri, USA	55	[56]

Since the beginning of the headstarting program in 2012 and the first release occurring in 2014, the population structure has become skewed towards juveniles. This is not surprising given that all released turtles are juveniles. Blanding’s Turtles typically reach sexual maturity at a minimum age of 14 years [57] but may take up to 20 years at northern latitudes (e.g., Nova Scotia) [58]. Interestingly, a headstarting program on Blanding’s Turtles in Illinois has reported reproduction at 11 years of age [12]. It is plausible that the accelerated growth rate in human care and thereby larger body size at release may influence the age at sexual maturity for headstarted turtles. Similar results have been reported for a Wood Turtle (*Glyptemys insculpta*) population in Ontario, Canada, where male headstarted turtles reached sexual maturity earlier than females [10]; however, females in this Wood Turtle population matured at the same age as wild-born turtles, albeit at a larger size. The size and age at which sexual maturity is attained in turtles is a complex phenomenon [59] requiring further research, and headstarting may impact this life-history relationship.

Blanding’s Turtles from our headstarting program have very high survival estimates (89%). Only one release cohort experienced unusually high mortality in 2019, when survival plummeted to 43% immediately after release. Mortality at our study site occurs mainly during spring, and we suspect that subsidized predators, such as Raccoon (*Procyon lotor*), Mink (*Neovison vison*), Red fox (*Vulpes vulpes*), and Coyote (*Canis latris*) are the primary source of mortality. However, in 2019 and 2020, we observed high mortalities of headstarted Blanding’s Turtles and adult female Painted Turtles; more than 50 individuals of each species were depredated during the nesting season (Toronto Zoo [Unpublished]). The mass mortality event likely negatively affected the 2019 release cohort more so than other cohorts because newly released headstarted turtles sometimes experience a behavioural acclimation period during which they display a reduced ability to identify and evade predators and navigate complex habitats [42]. A study on Snapping Turtles in Algonquin Provincial Park showed that a decline of 8–18% in adult survivorship can have detrimental effects on population abundance in the long-term, even if survivorship returns to pre-catastrophe levels [60]. To fully understand the effects of the mass-mortality event on the headstarted population, continued monitoring will be critical. In addition, estimates from population viability analyses should be re-evaluated to account for catastrophic events and to decide whether headstarting should be continued in the long-term, given the on-going conservation challenges at the study site. Annual survival estimates of headstarted Blanding’s Turtles vary depending on age and geography, and our results are similar to survival estimates reported elsewhere. For example, in a Michigan population, survival ranged from 63–96% [25], in Nova Scotia survival ranged from 70–80% [61], in an Ontario population, monthly survival ranged from 89–98% [42].

Given that the goal of headstarting is to increase the survival of hatchling turtles compared to wild ones, it is important to compare the survival of headstarted and wild Blanding’s Turtles of similar age. Unfortunately, we did not observe any wild-born hatchlings in the RNUP population, and only one direct-release hatchling was captured; therefore, we were unable to make a meaningful comparison. Two studies that investigated the survival of headstarted Blanding’s Turtles compared to wild ones found that both headstarted and wild turtles have similar survival rates despite headstarted turtles being larger in size [61, 62]. Although headstarting relies on the assumption that larger body size minimizes predation risk (i.e., bigger is better) [16, 63], the findings by Arsenault [61] and Golba et al. [62] suggested larger body size is not the only indicator of increased survival. For example, exposure time (e.g., active time spent from nest emergence to overwintering) and habitat use patterns are significant predictors of hatchling survival [64, 65]. In addition, use of soft-release, environmental enrichment, and antipredator training have positively influenced post-release survival in other animal taxa [66]. In light of current evidence, careful consideration must be given prior to undertaking headstarting programs as a population restoration strategy, because relying on the “bigger is better” hypothesis alone may not be sufficient to increase recruitment into the population in the long term.

The recapture probability of juvenile headstarted Blanding’s Turtles remained low throughout the study period, and was especially low after the 2019 mass mortality event. During trap surveys, we inspected traps once daily. Our recapture probability would likely increase if the traps were checked more frequently, as on at least three occasions, we observed juvenile Blanding’s Turtles escaping hoop traps. In addition, juvenile turtles are smaller in size, which makes it difficult to detect and capture them via other methods such as visual-encounter surveys [47].

### Sex ratio

The sex ratio in the RNUP population was equal, although wild populations often demonstrate a female-biased sex ratio [55, 67–70]. However, in most studies, the sex ratio reported is likely an artefact of biased sampling and not a true reflection of the population sex ratio. Sex of the headstarted turtles in our study was assumed based on incubation temperature; we cannot be certain whether the presumed sex is accurate until the turtles reach sexual maturity. Once the headstarted turtles reach sexual maturity, future studies should investigate whether there is differential mortality between the sexes. While the proportion of females was lower in the recapture sample compared to the proportion at release, this difference was not significant. Monitoring sex ratio is especially important for the long-term persistence of turtle populations because biased sex ratios are an early indicator of population decline and may result in lower effective population size and overall recruitment [71, 72].

### Conservation implications

Although headstarting has been used to augment the wild population in the RNUP, and the results appear favourable in terms of juvenile survival estimates and population sex ratio, headstarting alone is likely inadequate to ensure the long-term persistence of the headstarted population given the location and size of the release site, and urban nature of the study area. For example, all headstarted Blanding’s Turtles inhabit a small wetland complex in a highly fragmented landscape with high road density and low connectivity to other suitable habitats [21, 22]. Given that the RNUP Blanding’s Turtle population density is one of the highest reported, we are uncertain whether the release site has reached its carrying capacity and cannot support additional supplementation of headstarted turtles in the long term. In addition, the release site provides habitat to two other native turtles, Painted and Snapping turtles, increasing competition for basking, foraging, overwintering, and, later, for nesting sites when headstarted turtles reach sexual maturity. The limited habitat connectivity and high density of roads may be problematic because Blanding’s Turtles travel extensive distances to find mates and nesting habitats [68, 73, 74], which puts them at greater risk of vehicular collisions. Therefore, mitigating road mortality (e.g., installation and maintenance of fencing, speed bumps) and increasing habitat connectivity (e.g., eco-passages) will be crucial for ensuring the long-term persistence of the species in the RNUP.

Another potential challenge associated with the release site is that the ‘National Park’ status of the RNUP provides numerous, but limited, protections to the headstarted Blanding’s Turtles. The Rouge National Urban Park Act [28] provides a legal avenue to protect native turtles, other wildlife, and plants by prohibiting activities such as disturbance, removal, hunt, or harvest of fauna and flora within the park boundary. Given that protection applies to all flora and fauna in the park, implementation of predator-control initiatives to prevent future mass mortality events is a complex issue. In addition, other threats, such as agricultural practices, non-point source pollution (e.g., use of road salts in the winter), and recreational activities continue to directly and indirectly impact turtle populations. For example, a study by Garber and Burger [75] found that human recreation (e.g., hiking, fishing) resulted in extirpation of a Wood Turtle population at two sites within a 10-year study period, likely in part as a result of incidental take of turtles. Other studies have shown that population declines continue in protected areas despite habitat protection [10, 18, 72, 76]. Without proper management and enforcement, parks and conservation reserves do not fully protect at-risk species. The RNUP is also open to recreational activities and agricultural practices that may limit the ability to recover the resident Blanding’s Turtle population. Turtles in similar urban landscapes elsewhere inevitably face similar threats.

## Conclusion

The RNUP population of headstarted Blanding’s Turtles increased in abundance, showed high survival rates, and an equal sex ratio. Our findings are similar to those of headstarting programs elsewhere and add to the current knowledge of the efficacy of headstarting programs on a broader scale. However, to fully reach its recovery potential, headstarting should be integrated into comprehensive conservation plans that address root causes of population decline. The size and the location of our study area require several other conservation actions to be developed and implemented within a reasonable time frame. Continued monitoring of the headstarted population will be critical to evaluate the success of the headstarting program.

## Supporting information

S1 File Encounter history of the headstarted Blanding’s Turtles in the RNUP. (XLSX)

S2 File Parameter index matrix for the best model in Program MARK. (XLSX)

S3 File Complete results output of Jolly-Seber POPAN model ranking. (DOCX)
