# Supplementary material for: Evaluation of headstarting as a conservation tool to recover Blanding’s Turtles (Emydoidea blandingii) in a highly fragmented urban landscape
Source: PLoS One. 2023 Mar 8;18(3):e0279833. doi: 10.1371/journal.pone.0279833 (PMC9994698; doi:10.1371/journal.pone.0279833)
Supplement: S3 File — (DOCX) [file pone.0279833.s003.docx]

Table S3: Complete results output of Jolly-Seber POPAN model ranking. *Φ* and *p* refer to apparent survival and recapture probabilities, respectively for Blanding’s Turtles in the Rouge National Urban Park. ‘acc’ refers to the effect of acclimation with two levels: newly released headstarted turtles and those in their first year following release.

| **Model** | ***k*** | **AICc** | **ΔAICc** | ***w*** | **Deviance** |
| --- | --- | --- | --- | --- | --- |
| *Φ*(t+acc2019)*p*(t)^§^ | 9 | 3089.93 | 0.00 | 0.18 | 2894.26 |
| *Φ*(t+acc2019)*p*(t*acc)^§^ | 12 | 3090.83 | 0.90 | 0.11 | 2888.67 |
| *Φ*(t+acc2019)*p*(t+acc)^§^ | 12 | 3091.24 | 1.32 | 0.09 | 2889.08 |
| *Φ*(acc2019)*p*(t)^§^ | 5 | 3091.60 | 1.67 | 0.08 | 2904.37 |
| *Φ*(acc + t2019)*p*(t)^§^ | 6 | 3091.83 | 1.90 | 0.07 | 2902.51 |
| *Φ*(acc + t2019)*p*(t*acc)^§^ | 9 | 3091.89 | 1.96 | 0.07 | 2896.22 |
| *Φ*(acc + t2019)*p*(t+acc)^§^ | 9 | 3091.89 | 1.97 | 0.07 | 2896.23 |
| *Φ*(t*acc)*p*(t) | 12 | 3092.01 | 2.09 | 0.06 | 2889.85 |
| *Φ*(acc2019)*p*(t*acc)^§^ | 8 | 3092.07 | 2.14 | 0.06 | 2898.53 |
| *Φ*(acc2019)*p*(t+acc)^§^ | 8 | 3092.22 | 2.29 | 0.06 | 2898.69 |
| *Φ*(cohort)*p*(t) | 8 | 3092.98 | 3.05 | 0.04 | 2899.44 |
| *Φ*(t*acc)*p*(t*acc) | 15 | 3093.29 | 3.36 | 0.03 | 2884.48 |
| *Φ*(cohort)*p*(t*acc) | 11 | 3093.79 | 3.87 | 0.03 | 2893.81 |
| *Φ*(cohort)*p*(t+acc) | 11 | 3093.90 | 3.98 | 0.02 | 2893.92 |
| *Φ*(t)*p*(t) | 8 | 3094.85 | 4.92 | 0.01 | 2901.31 |
| *Φ*(t)*p*(t*acc) | 11 | 3095.01 | 5.08 | 0.01 | 2895.03 |
| *Φ*(t)*p*(t+acc) | 11 | 3095.63 | 5.70 | 0.01 | 2895.64 |
| *Φ*(2019)*p*(t*acc)^§^ | 8 | 3098.23 | 8.31 | 0.00 | 2904.70 |
| *Φ*(2019)*p*(t)^§^ | 5 | 3098.46 | 8.53 | 0.00 | 2911.23 |
| *Φ*(2019)*p*(t+acc)^§^ | 8 | 3098.57 | 8.64 | 0.00 | 2905.04 |
| *Φ*(t*acc)*p*(t+acc) | 15 | 3099.29 | 9.37 | 0.00 | 2890.49 |
| *Φ*(.)*p*(t*acc) | 7 | 3107.07 | 17.15 | 0.00 | 2915.66 |
| *Φ*(.)*p*(t) | 4 | 3107.81 | 17.89 | 0.00 | 2922.66 |
| *Φ*(acc)*p*(t*acc) | 8 | 3109.19 | 19.26 | 0.00 | 2915.66 |
| *Φ*(acc)*p*(t) | 5 | 3109.84 | 19.92 | 0.00 | 2922.62 |
| *Φ*(t+acc2019)*p*(acc)^§^ | 8 | 3112.22 | 22.29 | 0.00 | 2918.68 |
| *Φ*(t*acc)*p*(acc) | 11 | 3112.45 | 22.53 | 0.00 | 2912.47 |
| *Φ*(acc)*p*(t+acc) | 8 | 3112.86 | 22.93 | 0.00 | 2919.33 |
| *Φ*(t)*p*(acc) | 7 | 3112.91 | 22.98 | 0.00 | 2921.49 |
| *Φ*(t+acc2019)*p*(.)^§^ | 7 | 3117.82 | 27.89 | 0.00 | 2926.40 |
| *Φ*(t*acc)*p*(.) | 10 | 3118.36 | 28.44 | 0.00 | 2920.55 |
| *Φ*(t)*p*(.) | 6 | 3119.64 | 29.71 | 0.00 | 2930.32 |
| *Φ*(2019)*p*(acc)^§^ | 4 | 3121.32 | 31.39 | 0.00 | 2936.17 |
| *Φ*(cohort)*p*(acc) | 7 | 3121.72 | 31.79 | 0.00 | 2930.31 |
| *Φ*(acc2019)*p*(acc)^§^ | 4 | 3122.03 | 32.10 | 0.00 | 2936.87 |
| *Φ*(cohort)*p*(.) | 6 | 3125.20 | 35.28 | 0.00 | 2935.89 |
| *Φ*(.)*p*(acc) | 3 | 3139.50 | 49.57 | 0.00 | 2956.40 |
| *Φ*(acc)*p*(acc) | 4 | 3141.34 | 51.41 | 0.00 | 2956.18 |
| *Φ*(.)*p*(t+acc) | 7 | 3142.51 | 52.58 | 0.00 | 2951.09 |
| *Φ*(.)*p*(.) | 2 | 3143.38 | 53.46 | 0.00 | 2962.33 |
| *Φ*(acc2019)*p*(.)^§^ | 3 | 5959.40 | 2869.47 | 0.00 | 5776.30 |
| *Φ*(2019)*p*(.)^§^ | 3 | 5960.95 | 2871.02 | 0.00 | 5777.86 |
| *Φ*(acc)*p*(.) | 3 | 5979.70 | 2889.78 | 0.00 | 5796.61 |

§, *post hoc* models that were proposed based on initial results suggesting a specific 2019 acclimation effect on *Φ; k*, number of parameters; AIC_c_, Akaike’s Information Criterion with small sample correction; ΔAIC_c_, difference in AIC_c_ between a given model and the best supported model; *w*, Akaike weight
